# Treatment of Segmental Loss of the Tibia by Tibialisation of the Fibula: A Review of the Literature

**DOI:** 10.5812/kowsar.22517464.3184

**Published:** 2012-01-15

**Authors:** Alireza Rahimnia, Frank Fitoussi, George Penneçot, Keywan Mazda

**Affiliations:** 1 Trauma Research Center, Baqiyatallah University of Medical Sciences, Tehran, IR Iran; 2 Department of Orthopedic Surgery Robert-Débre Hospital, Paris, France

**Keywords:** Segmental, Fibula, Ilizarov Technique, Tibia

## Abstract

Segmental defects of the tibia are challenging therapeutic problems for both the physician and the patient. These defects may be caused by severe trauma, infection, tumors and congenital processes. Several different techniques have been described for treatment of these defects including the Papineau technique, allograft reconstruction, bone transport using the Ilizarov frame, free vascularized fibular graft, tibiofibular synostosis and medial transport of the fibula with Tuli’s technique, use of the Ilizarov frame and Huntington’s procedure. All of these techniques have their specific advantages as well as disadvantages. Some of these techniques are used rarely i.e. the Papineau technique. The procedure of choice for most large tibial defects is bone transport with Ilizarov’s technique; but in some cases the tibial remnant is inadequate for lengthening and we must use alternative treatments. In the three aforementioned techniques, the fibula is transferred with peroneal and anterior tibial muscles on a pedicle of peroneal vessels. This transfer retains a biological component of vital bone that allows for a shorter time for consolidation, increased remodeling potential and resistance to infection. It also has better long-term mechanical properties. Hypertrophy of the centralized fibula is described as attaining twice its original diameter or twice the size of the contralateral tibia. Hypertrophy has been seen in nearly all cases of the fibular centralization. Maximum hypertrophy is seen in children and besides patient age, is related to bony union and weight bearing. The reported time for hypertrophy of fibula varies from one to four years. No significant change in the diameter of the fibula was observed after five years. Fracture of tibialized fibula was not reported in many studies of fibular centralization with different techniques. In the reviewed articles, there were no cases of valgus deformity of the ankle. Either the patients were satisfied with the final results despite appearance of the lower extremity and the presence of some angular deformities, although in most cases, the deformities were mild. In this review we conclude that tibialisation of the fibula in selected cases is a reasonable alternative for the treatment of massive tibial defects.

## 1. Introduction

Segmental defects of tibia are problematic for both the physician and the patient. Four of the most common causes of these defects are: Congenital (i.e. tibial agenesis or pseudoarthrosis of the tibia), bone infections, high energy fractures and bone tumors.

### 1. 1. Congenital Tibial Deficiency

Tibial hemimelia is a preaxial longitudinal deficiency with variable degrees of tibial absence. In most affected children there is no known cause. However, there are autosomal dominant forms and at least one report of autosomal recessive inheritance. In inheritable forms, the involvement is usually bilateral and there is duplication of toes and the possibility of hand anomalies. Associated abnormalities include syndactyly, polydactyly, split hand and foot, five-fingered hand, anonychia, radioulnar synostosis, radial ray agenesis, foot oligodactyly, bifid femur, ulnar and fibular reduplication, micromelia, trigonomacrocephaly, joint hyperextensibility and deafness. In a retrospective review of a 22- year period , 79% of patients with tibial deficiency had associated congenital anomalies with abnormalities of the hip, hand or spine. (1, 2). Four distinct autosomal dominant syndromes have been identified: Tibial hemimelia with footpolydactyly-triphalangeal thumb syndrome (Werner’s syndrome), diplopodia, split hand and foot syndrome, and micromelia-trigonobrachycephaly syndrome (1, 2).

**Figure 1.**
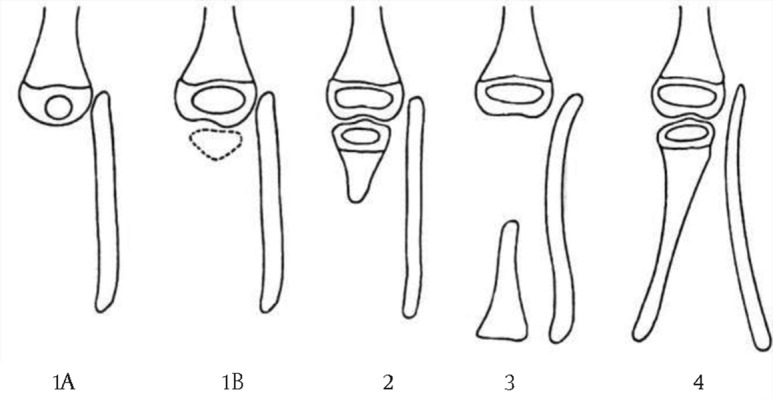
Jones’ Classification (1, 2)

The Jones system (*Figure 1*) classifies tibial hemimelia into four types based on radiographic features present during infancy. In type 1 the tibia cannot be seen on radiographs at birth. In subtype 1a the tibia is completely absent and the ossific nucleus of the distal femur is small or has not appeared. In type 1b the cartilaginous analogue of the tibia is present at birth but it is not ossified (but can be detected with ultrasonography or MRI) and the distal femoral epiphysis is normal. In type 2 the proximal part of the tibia is present at birth radiographically but the distal tibia is not seen. In type 3 the distal part of the tibia is present and ossified but the proximal portion of the tibia is absent. This type is the least common form. In type 4 the tibia is short and there is distal tibiofibular diastasis. In this type the articular surface of the distal tibia is absent and there is proximal displacement of the talus (1, 2).

### 1. 2. Infection of the Tibia

Another cause of tibial defects is bone destruction due to osteomyelitis itself or debridement surgery (that is fundamental in treatment of osteomyelitis, especially chronic osteomyelitis). The major goal of treatment in osteomyelitis is to remove the sequestrum, abscess cavities and granulation tissue that harbor bacteria and prevent the circulation of systemic antibiotics into the infected tissues. Enough bone and soft tissue must be resected to allow antibiotic therapy to complete the process. Extensive debridement, inadequate involucrum formation or pathologic fractures result in significant segmental defects (1, 2).

### 1. 3. Trauma

Open fractures particularly of the tibia are usually the result of high energy trauma, most commonly from automobile-pedestrian or automobile-bicycle accidents. Fractures associated with extensive soft tissue damage to muscles, skin and neurovascular structures and a high degree of contamination and bone comminution are major components of Gustilo type 3 open fractures. This severe comminution may cause a segmental defect. The risk of bone infection in open fractures is greater than closed fractures and can lead to bone loss (1, 2).

### 1. 4. Neoplasms

The diaphysis of long bones is the site of involvement of various benign and malignant tumors such as fibrous dysplasia, osteoblastoma, histyocytosis x, lymphoma, admantinoma and Ewing‘s sarcoma. In most of these tumors a wide restction margin is necessary for treatment. On the other hand limb-salvage procedures produce bone defects that must be reconstructed (1-3).

## 2. Techniques for treatment of tibial defects

### 2. 1. Papineau’s Technique

Open excision and cancellous bone grafting for treatment of chronic osteomyelitis is based on the following principles: Granulation tissue resists infection, autogenous cancellous bone grafts are rapidly revascularized, the infected area is completely excised, adequate immobilization is provided and antibiotics are used for prolonged period. Supplying only cancellous bone which has limited mechanical strength is the major disadvantage. A large amount of graft is not available in children. Full weight-bearing is started 11 months after surgery in those with a defect larger than 1 cm with the worst prognosis in patients with tibial defects; it requires a mean hospitalization of 3 months and nurses experienced in bone site irrigation technique (1, 4-6).

### 2. 2. Use of Allograft

Allograft reconstruction of bone defects is a well-known procedure especially after tumor resections. However implantation of large amounts of allograft has been associated with greater risk of infection, graft rejection, fracture, nonunion and fear of disease transmission (7-9).

### 2. 3. Bone Transport

The Ilizarov technique in treatment of defects (due to osteomyelitis, fractures or agenesis), involves bone transfer and compression-distraction to stimulate new bone formation. Early weight-bearing which reduces the risk of disuse osteoporosis and stiffness in adjacent joints is possible. A major advantage is the possibility to correct malalignment before or during bone transfer. The use of this technique provides different treatment options such as bifocal or trifocal bone transport with or without acute or gradual shortening to treat tibial defects. Earlier methods showed that distraction osteogenesis increases blood flow by 3 to 10 times in the extremity enhancing the local tissue level of antibiotics. The mean healing time of bony defects in patients with bifocal extension-distraction is 270 days and without it is 170 days. The major drawback of this technique is that it is not well tolerated by the patients. The most common complication is pin tract infection. In children the docking sites generally do not require bone grafting. Neurovascular injuries, muscular damage, articular injury, deep vein thrombosis, refractures, deformities and scarring are other complications of Ilizarov limb lengthening (1, 3, 5, 10-12).

### 2. 4. Free Vascularized Fibular Graft

Advances in microsurgical techniques have made preservation of the intrinsic vascularity of bone graft possible and use of the vascularized fibula graft became popular in filling bone defects. The fibular graft is harvested with a long pedicle of peroneal artery and vein. Free vascularized grafts live on their own vessels and do not undergo creeping substitution. They are also able to heal quickly and resist infection. Injury to the peroneal nerve must be avoided and adequate fibula length must be retained for ankle stability. The use of free vascularized graft from the contralateral extremity adds morbidity, is technically difficult and requires special expertise and equipment. On the other hand a satisfactory anastomosis may be difficult in case of infection and ankle instability and peroneal nerve injury may be disasterous (1, 3, 5, 6, 8, 9, 13-16).

### 2. 5. Tibiofibular Synostosis

This is done by placing a bone graft superior and inferior to the tibial defect between the tibia and fibula above the interosseous membrane. This method carries the risk of fibular fracture because of eccentric loading and in the lesions of the distal tibia the synostosis reduces the range of motion of the ankle (5).

### 2. 6. Medial Transport of the Fibula Using Various Techniques

**Figure 2.**
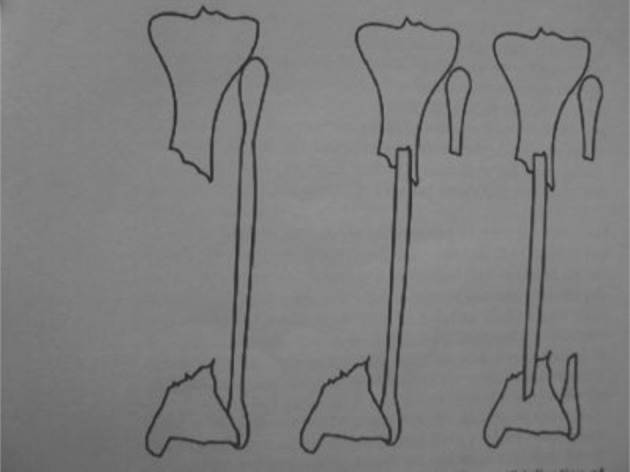
Tuli’s technique for medial transfer of the fibula

A: Tuli’s technique: Tuli operated 21 patients; 19 of them were between 18 and 43 years old and the other two were children who were 4 and 6 years old. All tibial defects were caused by fractures and infection. His patients had an intact fibulas, good vascularity and normal sensation of the sole of the feet. For patients with supple soft tissue he transferred the proximal and distal ends of the fibula to the tibia simultaneously in a trap door fashion;, but in patients with severe scarring especially in the lateral compartment of the leg he did medial transfer of the proximal fibula first and the distal end later 3-6 weeks (*Figure 2*). In 3 patients because of loss of distal end of tibia he transferred the distal end of the fibula to the talus first. The fibula was stabilized with either calcaneal pins, pins and screws or only screws. After each stage, a cast was placed for 3 weeks with out weight bearing; then a well molded cast was applied and weight bearing was encouraged for 5-8 months. All 21 tibializations resulted in synostosis between the tibia and fibula with three additional operations in three patients including screw exchange and bone grafting (6).

**Figure 3.**
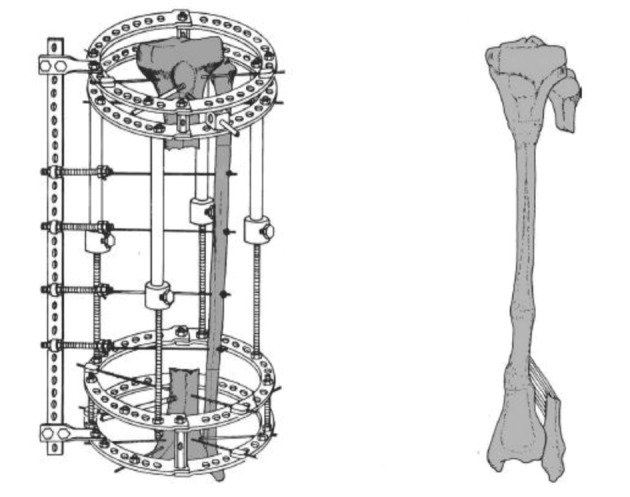
Medial Transfer of the Fibula via the Ilizarov Frame

B: Medial transport of the fibula with the Ilizarov frame: Catagni used the Ilizarov frame for medial transfer of the fibula in seven patients with massive trauma-related tibial defects from 7 to 28 centimeter aged between 23 and 63 years with massive tibial bone loss or poor residual bone not suitable for Ilizarov lengthening. Their other indications were to supplement the strength of lengthened tibia. He did not exclude patients with fractured fibulas. Prerequisites were acceptable blood supply and normal sensation of the plantar surface of the foot. They placed one or two distal and proximal rings. Osteotomies were done according to the tibial defect and olive wires were placed on the lateral aspect of the fibula from posterolateral to anteromedial to direct the fibula to the posterior aspect of the tibia (*Figure 3*). Seven days after osteotomy, transport of the fibula was started at a rate of 0.25 mm each six hours. Bone graft was used at four docking sites in two patients because of nonunion after fibular transfer or to augment union and bone formation. The patients had weight bearing throughout treatment; the frame was removed after consolidation and then a cast was applied (10).

**Figure 4.**
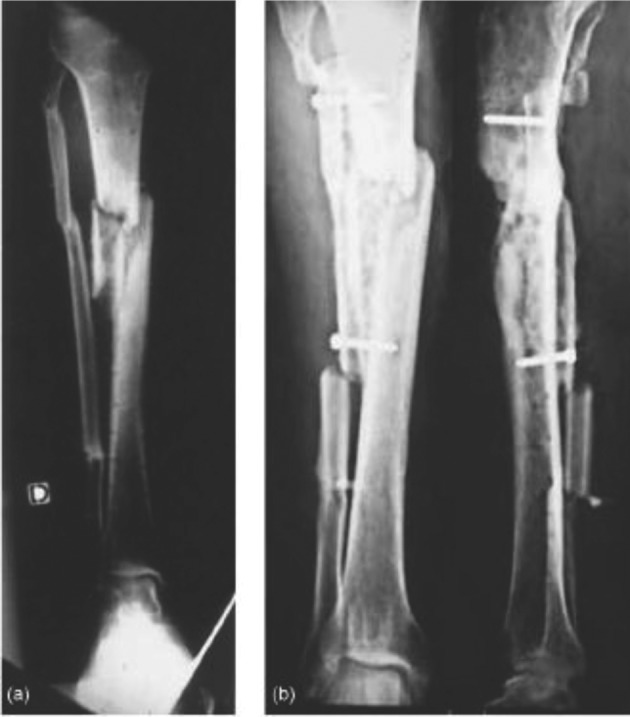
Huntington Technique of Medial Transfer of Fibula

C: Huntington procedure: Cassab did 11 cases of ipsilateral fibular transposition in patients aged between 16 and 61 years for tibial defects from 4 to 22 cm caused by severe trauma, osteomyelitis or tumor resection. They recommended arteriography for assessment of lower limb vasculature and to select the best surgical approach. They used a posterolateral approach to the leg and in the case of anterior tibial artery injury they used an anterolateral approach. Fistulas with external opening to the skin were excised whereas internal fistulas were left intact. In the next step, the tibia was decorticated with distance from the nonunion site for avoiding exacerbation of possible infection. After that they did osteotomy of the fibula and after medialization of the fibula, screws were used to fix the fibula to the tibia and bone grafts were used only at the junction of the tibia and fibula (*Figure 4*). Immobilization was done by cast or external fixator and weight bearing was not allowed until union of the tibiofibular junction. Healing was achieved in 8 patients. Three patients required additional procedures with final union. In two patients amputation was done because of intractable infection (4, 11).

## 3. Results of various techniques

Ilizarov corrected defects of the tibia caused by infection and trauma; the time to transport and union ranged from 6 to 18 months. The amount of tibial defect ranged from 7 to 28 cm and the time required for using a walking assist after frame removal ranged from 1 to 65 months. All patients had hypertrophy of the fibula and satisfactory joint motion. Two pin tract infections were treated by local pin site care and oral antibiotics. At 5-year follow up two of the seven patients walked wearing orthoses for protection of the leg from trauma. All of patients were adults (10). However, in another study of fibular transfer in two children because of fracture and infection with defects of 11 and 12 cm, the time for transfer and retaining Ilizarov device was 2.5 to 4 months with protection and an additional one to two months thereafter. At two and three years postoperatively there was hypertrophy of the fibula (5). Medial transfer of the fibula by Ilizarov frame also was used for the reconstruction of a 15 cm defect of the tibia in a 41 year-old. The cause was a motorcycle accident. After one year and 10 days the frame was removed after union and consolidation of the two ends (17).

In a study of 21 patients (19 adults and 2 children) with tibial defects by Tuli, hypertrophy of the fibula occurred in all patients and the size almost doubled in four years after the success of synostosis and there was no significant change in the diameter of the fibula after five years; and finally remodeling restored the continuity and size of the medullary canal (6). In other similar methods of treatment of tibial defects caused by bone tumor resection in 13 patients with mean age 17 years (range 7-40) which were between 10 to 21 cm, ipsilateral fibular transfer and fixation was done via a combination of plates, screws and Kirschner wires. None of the junctions were grafted. Aside from common peroneal nerve injury in one patient with complete recovery no other immediate postoperative complication was seen. Twelve patients had junctions which were completely united. One patient had hypertrophic nonunion of distal junction and declined further surgery, and walked with the aid of a cane (6). In another patient with hypertrophic nonunion of the distal junction an Ilizarov device was used and finally union was achieved. The mean time to union at the junctions was 12 months varying from 3 to 36 months (3). In an other report a congenital pseudoarthrosis of the tibia in a boy was treated with ipsilateral transfer of the fibula. At first (age 2 years 7 months) he was treated with Sofiled-Millar operation augmented with cortical bone from the normal side; however, the bone resorbed rapidly. The contralateral fibula was split longitudinally and inserted in the absent tibia and supplemented with iliac crest bone grafts at the age of 2 years 10 months. It also resorbed. Finally, he underwent transfer of ipsilateral fibula with tibiocalcaneal intramedullary pin. At one year postoperatively the fibula had united with the tibia. The fibula gradually hypertrophied but the patient had to use a protective brace and then ankle foot orthosis until age 17 years (9). In a similar study, 16 infected nonunions of the tibia with an average defect size of 6.12 cm were treated with transfer of the ipsilateral tibia to the posterior aspect of the tibia. The graft was fixed with cancellous screws or intramedullary pins adding bone graft at bone ends. In all 16 patients union occurred within a minimum time of 6 weeks in children and 1.5 years in adults. Hypertrophy of the fibula was seen in 15 patients within one to two years (17). In the study of 11 patients with medial transfer of the fibula according to the Huntington technique healing was achieved in 8 patients within a mean time of 10.5 months (7 to 22 months). After around 10.5 months it was strong enough to allow weight bearing (11). In many patients of aforementioned studies the range of motions of the knee and ankle after union of the fibula to the tibia was normal. There were some degrees of restriction in a few patients with mild ankle deformities (equinus or hind foot varus). 

## 4. Discussion

Large segmental defects of the tibia are challenging therapeutic problems (1, 2, 4, 5, 11, 13). They can be caused by severe trauma, osteomyelitis, tumor resection, reconstruction or congenital etiologies such as agenesis or pseudoarthrosis of the tibia. Various methods of treatment have been used to treat such cases including Papineau procedure, allograft reconstruction, distraction osteogenesis , vascularized or nonvascularized contralateral fibular transfer and tibiofibular synostosis. Each of these techniques has its specific advantages and disadvantages.

Albert first proposed the use of the fibula as an alternative for the tibia in 1877. He obtained fusion between the fibula and femur in a patient with congenital absence of the proximal tibia. In 1884 Hanh used ipsilateral fibular grafts to treat massive tibial bone loss. The transfer of the ipsilateral fibula is done with peroneal and anterior tibial muscles and pedicle of peroneal vessels. Huntington used arteriography before transferring the fibula medially; others did not use or recommend it. The main advantage of this transfer is retaining biological potential of living bone that allows for a shorter time for consolidation, increases remodeling potential and resistance to infection and also has better long-term mechanical properties (8, 18, 19). Kassab *et al* did angiography 6 and 8 weeks after fibular transfer and found a normal pattern of peroneal artery and muscular-periosteal network. Puri emphasized retaining vascularity of the transferred fibula. In his series of ten junctions that needed a second operation, six developed hypertrophic nonunion. This suggests that the transposed fibula retained its biological potential but lacked stability (3).

The range of time for union of the fibula to the tibia in reported series is 6 weeks (17) to 36 months (3). The shorter times were seen in children and longer periods in adults. The rate of bone healing decreases from infancy to skeletal maturity, however, after that there is no significant decrease in bone healing. One possible reason for the greater potential of union in children may be increased availability of cells that produce tissue repair (20). Stable internal fixation is the key point in achieving union; and Puri *et al*. stated that fixation with long plates and several screws is better than simple osteosynthesis with pins, when possible (3).Kassab *et al*. suggested that fibular transposition should be considered earlier. Multiple previous procedures may compromise blood supply and lessen the chance of union. He also considerd corticocancellous autografts for induction of earlier healing (11). Puri *et al*. used autogenous cancellous grafts or demineralized bone matrix at two ends of the transfer if he suspected that post-operative radiotherapy may be necessary (3).

Hypertrophy of centralized fibulas (described as reaching double its original diameter or the size of the contralateral tibia) is seen in nearly all of cases of fibula centralization. Maximum hypertrophy is seen in children and besides patient age, is related to bony union and weight bearing (17). The reported time for hypertrophy of the fibula varies from one to two years or from two to four years; however, Tuli *et al*. stated that no significant change in the diameter of the fibula is observed after five years (6, 17). Fracture of tibialized fibula was not reported in most studies of fibular centralization with different techniques (3, 5, 6, 9-11). Keeting *et al*. reported one stress fracture of the fibula in 16 cases of fibular transfer that were treated with sufficient immobilization time. Proximal migration of an unsupported lateral malleolus can cause ankle valgus, a well-known occurrence in young children. The ankle valgus is treated with fusion of the remaining distal fibula to the distal tibial metaphysis by one of several techniques. In the reviewed articles, there were no cases of ankle valgus deformity .In this review we conclude that tibialisation of the fibula in selected cases is a reasonable alternative for the treatment of large tibial defects. 
